# Inter-area minimisation of reactive power flow for voltage improvement in large electric grids

**DOI:** 10.1038/s41598-026-44284-z

**Published:** 2026-03-18

**Authors:** Manohar Singh, Wilis Negi, Vinay Kumar Jadoun

**Affiliations:** 1 Department of Electrical Engineering, Punjab Engineering College (Deemed to Be University), Chandigarh, 160012 India; 2 Manipal Institute of Technology, Manipal Academy of Higher Education, Manipal, India

**Keywords:** Artificial loading, Capability curve of generators, Dynamic reactive power, Short circuit strength, Transmission losses, Energy science and technology, Engineering

## Abstract

Traditionally, Electric grids are designed to transport the bulk power generated at conventional power plants to load centres. Electricity regulators are tightening the grid codes to improve the performance and efficiency of electric grids. The coordinated planning of reactive power management for voltage profile regulation is an essential aspect of the seamless operation and control of electric grids. The adverse effects of seasonal variation in load profiles and the de-commitment of generating units based on their viability magnify the voltage regulation challenge in the large electric grid. These operational scenarios create low-voltage pockets in the distribution network, and there is a high draw of reactive power from the upstream transmission system. The power transfer capabilities of inter-regional tie lines decrease, and subsequently, this leads to overloading of power equipment and increases active loss in the utility grid. In this research article, reactive power management is formulated as a nonlinear optimisation problem. The objective function is minimised as the sum of reactive power flow over inter-intra-regional tie lines in a large electric grid. The optimisation problem is solved with a set of constraints imposed by placing the local reactive power support devices at critical locations. Selection of critical locations is identified through a new hybrid voltage-grid strength sensitivity index. The proposed index utilises the grid strength in addition to the voltage sensitivity of bus selection of buses for the installation of reactive power support devices. The performance of the proposed algorithm has been tested on real data from the Northern region of the Indian grid, which includes seven power transmission utilities with over 9000 buses. The simulation results show that the proposed reactive power optimisation, based on the hybrid voltage-grid strength sensitivity index, reduces reactive power import from 1592 to 383 MVAr by injecting 9421.8 MVAr at 33 kV at only 14.1% of the highly sensitive buses identified through this index. It is observed that injecting compensation devices at 33 kV buses improves the average bus voltage to nearly 0.98. The reactive power in the inter-intra tie lines decreases by 76%, and active power losses are reduced by 7.99%. In conclusion, the proposed algorithm can serve as a guiding tool for planning reactive power management in large-scale grid utilities, aiding voltage improvement with the optimal installation of compensating devices at a minimal number of 33 kV buses.

## Introduction

### Motivation

Electric grids are designed to transport the bulk electric power over long distances from generating sources to load centres. The grid operators schedule the active power for seamless operation, control, and high reliability of the electric grids^1^. The highly inductive nature of the electric grid consumes reactive power through its transportation, and this component of power is non-schedulable. Synchronous generating units of generation companies^2^ are operated in quadrants I and II to supply and absorb reactive power as needed in the grid, depending on the grid voltage profile. So, the adequate balance and management of reactive power in an Electric grid is very crucial for its efficient and reliable operation^3^. The large-scale utility grid typically encompasses more than one region, and these regions are further divided into multiple independent Power Transmission Companies (TRANSCO) grid operators to serve their respective Distribution Companies (DISCOM)^4,5^. Diversity in loading patterns, lack of power infrastructure, and grid operation practices may lead to indiscipline in their operation. This leads to the creation of low-voltage zones, excessive flow of unscheduled reactive power tie lines, and overload of power infrastructure. The effect is further propagated to inter-TRANSCO tie lines, and this finally leads to artificial loading of inter-regional tie lines in the National grid. The transmission utilities are penalised by grid regulators for the unscheduled exchange of reactive power. The active power flow on these tie lines is generally kept at the scheduled level. Contrary to this, unscheduled flow of reactive power leads to artificial loading of the Electric grid and increases the active losses, poor voltage regulation, load congestion, etc.^6,7^.

### Literature review

The DISCOM generally operates transformer taps or installs the reactive power compensating devices^8^ close to the load buses to avoid the long routing of unscheduled flow of reactive power. These devices are fixed capacitor banks, dynamic compensation devices, SVC, and even STATCOM at the industry point of connection^9^. The location of these devices is very crucial for their performance for the local control and management of reactive power in the DISCOM. However, the management of reactive power becomes very tricky and complicated in a National Grid comprising multiple regions and power utilities interconnected. The fundamental reason behind it is a lack of coordination and improper planning, different operational practices^2^, and diversity in load profiles in different power utilities in the highly diversified National Grid. The unscheduled flow of reactive power over tie lines attracts penalties to TRANSCO as per the grid regulations framed by the grid regulators.

Adequate reactive power support is essential for maintaining acceptable voltage levels in power systems. Insufficient VAR availability can initiate voltage instability, which may escalate into voltage collapse and ultimately cause widespread outages. Therefore, optimal allocation of new reactive power sources is a critical component of secure system planning. Existing research generally classifies reactive power planning methods into two major groups: index-based and optimisation-based approaches. Index-based approaches rely on sensitivity indicators and weak-bus ranking to reduce the search space for potential VAR installation sites, whereas optimisation-based methods employ mathematical programming or heuristic algorithms to determine the optimal reactive power placement and sizing. A detailed literature review on various index-based methods is presented in this research article. The different voltage sensitivity analysis methods are reported in the literature^10,11^ to compute the deviations in bus voltages for the expected change in power. These techniques in the existing literature can be broadly classified as fitting approaches^12^, which work in offline power flow analysis to determine the best fit for an assumed functional relation between the voltages and power levels. The fitted-based technique is computationally intensive and may not be suitable for the real-time application of large-sized power systems. Online monitoring^10^ and operation sensitivity factors are used as linear approximations either on the network side or on the nodal power injections side. This linearises the voltage sensitivities and reduces the computational effort. A low complexity voltage sensitivity^13^ analysis technique was developed that exploits an approximately linear relation between voltage change and power injection changes without compromising on voltage magnitude estimation accuracy.

Identification of critical buses in an electric grid is crucial for providing insight into identifying the weakest and most voltage instability-vulnerable buses^14^. Information about the venerable buses will enable the grid operator to take appropriate preventive measures by injecting reactive power support devices and continue to enhance the voltage stability margin^15,16^.

Different voltage stability indices have been proposed by several authors^17^. A simplified voltage stability index has been reported in the literature^18^. The index utilises voltage phasor measurements and configuration of the power system to determine the voltage stability margin. Measurement-based LM sensitivity methods can avoid complex calculations and reduce computational time. Voltage instability occurs when the voltage stability index breaches the critical limit in a bus. In^19^, a voltage stability index based on power flow equations has been developed. This voltage-sensitive index has been framed using the magnitudes of bus voltage and current to calculate the distance between the current operating point and the point of voltage instability.

The Voltage collapse prediction index has been reported in^20^ to predict voltage collapse in a power system. The technique is based on system variables such as the bus voltage angle, voltage magnitude, and admittance matrix of the system. The sensitivity analysis exploring the singularity of the Jacobian matrix for voltage stability assessment has been discussed^11,21^. An eigenvalue technique^22^ for determining the Jacobian at the maximum load equilibrium point for exploring the load margin has been discussed in^23^. The voltage sensitivity indices focusing on the weak voltage buses for the location of compensating devices have been discussed^24,25^.

Reactive power and VAR planning, as well as voltage control in interconnected transmission systems, increasingly emphasise inter-area coordination to minimise reactive power flows between regions, thereby improving voltage profiles and reducing stress on transmission corridors^26^. Traditional local VAR compensation often fails to address systemic issues, motivating optimisation across regions. Hybrid intelligence approaches, such as multi-load level reactive power planning using VAR compensators, have been discussed^27^ to integrate operational costs, installation costs, and transmission losses, enabling trade-offs between stability and efficiency. VAR reserve management and multi-objective dynamic VAR planning to maintain voltage stability under both steady-state and transient conditions have been well discussed in the literature^28,29^. Meta-heuristic optimisation techniques, including oppositional Harris Hawk^30^, Tabu Search^31^, and Chaotic Bat algorithms^32^, have demonstrated improved voltage profiles, reduced losses, and enhanced system reliability by optimally allocating reactive resources.

Recent advances focus on dynamic conditions, renewable integration, generator capability constraints, and short-circuit strength. Adaptive voltage control using local voltage profiles and similarity ranking^33^ had been applied for emergency response. Clustered effective reactive reserve indicators^34^ techniques are developed for vulnerable voltage zones identification in large electric grids. Allocation of Novel devices, including power-flow-control transformers with extended reactive support ranges for enhancing inter-area voltage management, has been reported in literature^35^. Dynamic reactive power dispatch, coordinated with inverter-based resources and FACTS devices for reduction in active power losses within generator capability curves and voltage limits, has been discussed^36,37^.

Traditional power systems are dominated by synchronous generators; the voltage support strength can be assessed by the short circuit ratio (SCR)^38^. The SCR is defined as the ratio of the three-phase short-circuit capacity of the connecting bus to the capacity of the connected device. SCR indirectly indicates the resiliency of the grid to maintain its voltage constant during the injection of active and reactive power^39^. During an extensive literature review, it is concluded that index-based methods to trap local minima and optimisation-based approaches are very effective for optimising the reactive planning problem^40^. Conventional mathematical optimisation approaches—such as successive linear programming^41^, mixed-integer nonlinear programming, and branch-and-bound have been applied to reactive power planning but often suffer from poor scalability and difficulty capturing the problem’s nonlinear, nonconvex nature. As a result, these approaches may converge to local rather than global optima. To address these limitations**,** meta-heuristic algorithms have become widely used due to their robustness, flexibility, and strong global-search capability. A comprehensive literature review on meta-heuristics for reactive power planning using shunt capacitors and FACTS devices has been conducted. Algorithms such as genetic algorithms(GA)^42^, evolutionary programming (EP)^43^, particle swarm optimisation (PSO)^44^, differential evolution(DE)^45^, ant colony optimisation (ACO)^46^, gravitational search algorithm(GSA)^48^ and Grey wolf optimisation algorithm (WOA)^49^ are applied for solving the reactive power dispatch problem.

Multi-objective optimisation^50^ has also gained focus for solving the reactive power dispatch problem for voltage improvement. Application of game theory for reactive power and voltage control in a power grid is also reported in the literature^51^. The objectives are typically framed as minimisation of real-power losses and investment costs**,** as well as maximisation of voltage stability margin**.** Voltage-stability indicators such as the local bus stability index(L-index), fast voltage stability index, line stability index^52^ and nodal line stability index^53^ are frequently used to evaluate candidate locations for installation of compensating devices for control of reactive power.

Recent studies emphasise the growing role of advanced optimisation techniques in enhancing the performance and resilience of modern power systems. In^54^, a stochastic economic framework integrates flexi-renewable virtual power plants and electric springs to improve smart distribution network efficiency. Research presented in^55,56^ focuses on resiliency-constrained planning approaches for optimal virtual power plant placement under extreme weather conditions. Similarly, Zadehbagheri et al.^57^ proposes stochastic sizing and siting strategies for hybrid hydrogen–power energy systems to support active distribution networks. In^58–60^, this research is further extended by introducing reliability-oriented transmission expansion planning using joint forecasting of loads and renewable energy generation.

Based on the extensive literature review, it is observed that reactive power planning research emphasises achieving an optimal trade-off between economic efficiency and enhanced system voltage stability, with meta-heuristic optimisation providing superior performance for large-scale grid applications.

The above-reported literature review is compared with the proposed research article in terms of main contribution, methodology adopted, research gaps and grouped in different clusters as summarised in Table 1 for the interest of readers. T

**Table 1:** A brief description of the literature.

Cluster	Refs.	Main contribution	Methodology	Research gap
Minimisation of tie-line reactive power flow in electric grids	Proposed	Minimisation of tie-line power flow with the installation of compensation devices at high-voltage sensitive and weak short-circuit buses to avoid reactive power penalties on power utilities	Reactive power is formulated as a non-linear optimisation with a set of constraints identified through a hybrid voltage-short circuit sensitive bus	The proposed problem is not extended to the renewables-rich electric grids. Limited supply of reactive power from renewable supplies in a large electric grid may have additional challenges
Reactive power management in DISCOMs	^2,8,9,26^	Discuss DISCOM-level reactive power management (capacitors, SVC, STATCOM) and challenges in multi-region national grids	Descriptive analysis of operational practices; inter-area VAR coordination studies	No integrated multi-area optimisation framework; limited treatment of uncertainty and dynamic conditions
Voltage sensitivity & weak-bus identification methods	^10–16,24,25^	Identification of weak buses, voltage change estimation, and location of reactive compensators	Offline fitting-based sensitivity, linearised online sensitivity, weak-bus ranking	Fitting-based methods are computationally heavy; linear methods are inaccurate under stressed nonlinear conditions; weak real-time capability
Voltage stability indices & collapse prediction	^11,17–23^	Development of voltage stability indices, Jacobian-based collapse indicators, and eigenvalue-based margin estimation	Voltage phasor-based indices, power-flow indices, Jacobian singularity, eigenvalue analysis	Computationally expensive for large grids; accuracy dependent on measurement quality; not robust under renewable variability
VAR planning, reserve management & multi-objective optimisation	^27–29,50–53^	Multi-load-level VAR planning, dynamic VAR reserve assessment, multi-objective optimisation (loss, cost, stability)	Multi-criteria optimisation, dynamic VAR planning, game theory, stability indices (L-index, FVSI, LSI, NLSI)	Limited modelling of uncertain renewable generation; reactive reserve modelling is incomplete; it lacks real-time applicability
Meta-heuristic optimisation for VAR planning and RP dispatch	^30–32,40–49^	Application of meta-heuristics for optimal VAR allocation, reduction of losses, and improved voltage stability	Harris Hawk, Tabu Search, Chaotic Bat, GA, EP, PSO, DE, ACO, GSA, WOA	Sensitive to parameter tuning; risk of local minima; high computational burden; limited scalability to large national grids
Advanced devices & inverter-based reactive support	^33–39^	Adaptive voltage control, reactive reserve indicators, power-flow-control transformers, and dynamic VAR with inverter-based generation	Similarity ranking, clustering, FACTS coordination, SCR-based strength evaluation	SCR is inadequate for inverter-dominated grids; dynamic SCR is not considered; there are high communication demands for coordination
Stochastic, robust & resilience-oriented grid planning	^54–60^	Stochastic planning integrating VPPs, electric springs, hydrogen systems, and renewable forecasting; resiliency-constrained expansion planning	Stochastic programming, robust planning, forecasting-based expansion frameworks	Reactive power aspects are rarely incorporated; focus on energy and generation, but not voltage stability or VAR margin

### Research gaps

The methods reported in the literature are broadly presented in this subsection. Placement of reactive power compensating devices at highly voltage-sensitive buses. The flow of reactive power on tie lines with other TRANSCOs within an electric grid is not considered as one of the parameters for managing the reactive power exchange. The grid regulations are in practice to ensure the power factor at the utility point of connection within 0.98 (lag/lead) and voltage deviation within 3% from the nominal value. The flow of reactive power exchange due to violations in power factor and voltage limits attracts penalties on TRANSCO utilities by grid regulators. The literature-reported methods do not emphasise the regulation of tie line flow for the management of reactive power. Additionally, the performance of the voltage sensitivity-based method for planning reactive power in a larger electric grid with multiple TRANSCO utilities is not addressed^28,29^.

To address these operational challenges, the grid operators are looking forward to a coordinated reactive power management approach for the Electric grid consisting of multiple power utilities.

### Contributions

In this research article, the reactive power management problem is formulated as a nonlinear optimisation problem. The minimisation of unscheduled flow of reactive power over tie lines in the electric grid is the primary objective. The reactive power compensation devices have been planned at minimal highly sensitive buses identified through a hybrid voltage sensitivity and grid strength index^8^.

The performance of the proposed nonlinear reactive power management problem is studied for the NR-region of the Indian Grid, consisting of multiple TRANSCO utilities with highly diverse load profiles. As a major outcome of this algorithm, it is observed that intra- and inter-regional flow of unscheduled relative power on the tie-line is minimised through the installation of reactive power injection devices at a few locations identified through the $HVGSSI$. The main contribution from this research article can be summarised as follows:

- **Minimisation of tie-line exchange of reactive power:** The uncontrolled flow of reactive power is minimised by formulating the reactive power flow problem as a nonlinear optimisation problem. The objective function is the sum of reactive power exchange across regional and interstate utility tie lines. It is observed that the proposed algorithm has resulted in an overall 76% reduction in reactive power import in the NR region from other regions of the Indian Grid. This has resulted in an average 8.0% reduction in active power losses within transmission utilities in the NR region**.**
- **Hybrid voltage—and grid strength sensitivity index**: This research paper proposes a $HVGSSI$ for identifying the minimum number of buses for reactive power support. Unlike methods based solely on the voltage sensitivity index, which perform poorly in large networks. These methods provide a large no. of buses, around 40% of the total buses, as candidate locations for reactive power support. Planning the reactive power support devices at such large locations becomes practically infeasible due to cost, operational challenges and maintenance reasons.
  The proposed $HVGSSI$ combines grid strength with voltage sensitivity and reduces the search space by identifying a small set of highly sensitive buses, about 15% of total buses, which are most suitable for installing compensation devices.
- **Potential for practical implementation in power utilities**: It is learnt that presently utilities plan reactive power support devices purely based on operational voltage experience & voltage sensitivity, and these methods require reactive power support at a large no. of buses, which is practically not feasible for them to implement. The proposed algorithm identifies a few buses for injecting the reactive power and will be a guiding method of planning reactive power support devices for a large-sized electric grid. The results presented in this paper are practical and are implemented in the NR region of the Indian grid. The method presented in this article will be a guiding method for power utility engineers for planning their reactive management in their electric grid.

### Paper organization

The research article is organised into four sections. Section “Introduction” deals with the introduction and literature review. Problem formulation is presented in Sect. “Problem formulation”. Section “Simulation & result” gives detailed results supported by discussion, and a conclusion is provided in Sect. “Conclusion”.

## Problem formulation

The generating units supply the reactive power support to match the requirements of transmission utilities based on their capabilities. Additional line transmission lines also contribute through line charging. But heavy reactive inductive demand from distribution size exhausts the inherent reactive power supply sources in transmission utilities, and they start withdrawing high Q over their tie lines. Power utilities are penalised for drawing the unscheduled exchange of reactive power from outside their areas. So, it becomes very crucial to manage the reactive power requirement locally within a transmission utility on their corresponding distribution buses for better voltage regulation^6,14,28^. The central idea of this problem formulation is the minimisation of bulk exchange of reactive power from the inter-transmission utilities’ tie lines connecting in the NR region with the National Grid. Identification of highly sensitive locations for installation of a minimum quantum of reactive power compensating resources is a very crucial aspect for local management of reactive power injection. An objective function is proposed that will minimise the exchange of reactive power flow on the tie lines. Because of this, the reactive power management problem in a large electric grid is formulated as a nonlinear optimisation problem. The proposed problem is addressed as follows:

### Power flow equations

The injected power equations are non-linear and are functions of bus voltage and power angles as listed in Eqs. (1–3).

1 $$P_{i} = \sum\nolimits_{k = 1}^{n} {|V_{i} \left| {\left| {V_{k} } \right|} \right|Y_{ik} |\cos \left( {\theta_{ik} + \delta_{k} - \delta_{i} } \right)}$$

2 $$Q_{i} = - \sum\nolimits_{k = 1}^{n} {|V_{i} \left| {\left| {V_{k} } \right|} \right|Y_{ik} |\sin \left( {\theta_{ik} + \delta_{k} - \delta_{i} } \right)}$$

These power flow equations are solved with the help of Newton-Raphson’s power flow iterative method^33,34^. The residual vector in terms of mismatch in active and reactive power, solution vector of bus voltage, and power angle for load buses and voltage-controlled buses are given in Eq. (4). The Jacobian element for change in active power and reactive power with power angle and bus voltage is listed in Eqs. (4–6) as under.

3 $$\left( {\begin{array}{*{20}c} {\begin{array}{*{20}c} {\Delta P_{i} } \\ {\Delta Q_{i} } \\ \end{array} } \\ \vdots \\ {\Delta P_{m} } \\ \end{array} } \right) = \left( {\begin{array}{*{20}c} {\begin{array}{*{20}c} {\frac{{dP_{i} }}{{d\delta_{k} }}} & {\frac{{dP_{i} }}{{d|V_{k} |}}} \\ {\frac{{dQ_{i} }}{{d\delta_{k} }}} & {\frac{{dQ_{i} }}{{d|V_{k} |}}} \\ \end{array} } & \ldots & {\begin{array}{*{20}c} {\frac{{dP_{i} }}{{d\delta_{m} }}} \\ {\frac{{dQ_{i} }}{{d\delta_{m} }}} \\ \end{array} } \\ \ldots & \ldots & \vdots \\ {\begin{array}{*{20}c} {\frac{{dP_{i} }}{{d\delta_{k} }}} & {\frac{{dP_{i} }}{{d|V_{k} |}}} \\ \end{array} } & \ldots & {\frac{{dP_{i} }}{{d\delta_{m} }}} \\ \end{array} } \right)\left( {\begin{array}{*{20}c} {\begin{array}{*{20}c} {\Delta \delta_{k} } \\ {\Delta |V_{k} |} \\ \end{array} } \\ \vdots \\ {\Delta \delta_{m} } \\ \end{array} } \right)$$

4 $$\frac{{dP_{i} }}{{d\delta_{k} }} = \left\{ {\begin{array}{*{20}c} { - \sum\nolimits_{\begin{subarray}{l} k = 1 \\ k \ne i \end{subarray} }^{n} {\left| {V_{i} } \right|\left| {V_{k} } \right|\left| {Y_{ik} } \right|\sin \left( {\theta_{ik} + \delta_{k} - \delta_{i} } \right), } } & {i = k} \\ {\left| {V_{i} } \right|\left| {V_{k} } \right|\left| {Y_{ik} } \right|\sin \left( {\theta_{ik} + \delta_{k} - \delta_{i} } \right), } & { i \ne k} \\ \end{array} } \right.$$

5 $$\frac{{dP_{i} }}{{d|V_{k} |}} = \left\{ {\begin{array}{*{20}c} { - 2\left| {V_{i} } \right|\left| {Y_{ii} } \right|\cos \theta_{ii} - \sum\nolimits_{\begin{subarray}{l} k = 1 \\ k \ne i \end{subarray} }^{n} {\left| {V_{k} } \right|\left| {Y_{ik} } \right|\cos \left( {\theta_{ik} + \delta_{k} - \delta_{i} } \right)} } & {i = k} \\ { - \left| {V_{i} } \right|\left| {V_{k} } \right|\left| {Y_{ik} } \right|\cos \left( {\theta_{ik} + \delta_{k} - \delta_{i} } \right), } & {i \ne k} \\ \end{array} } \right.$$

6 $$\frac{{dQ_{i} }}{{d\delta_{k} }} = \left\{ {\begin{array}{*{20}c} {\sum\nolimits_{\begin{subarray}{l} k = 1 \\ k \ne i \end{subarray} }^{n} {\left| {V_{i} } \right|\left| {V_{k} } \right|\left| {Y_{ik} } \right|\cos \left( {\theta_{ik} + \delta_{k} - \delta_{i} } \right) } } & { i = k} \\ { - \left| {V_{i} } \right|\left| {V_{k} } \right|\left| {Y_{ik} } \right|\cos \left( {\theta_{ik} + \delta_{k} - \delta_{i} } \right),} & {i \ne k} \\ \end{array} } \right.$$

7 $$\frac{{dQ_{i} }}{{d|V_{k} |}} = \left\{ {\begin{array}{*{20}c} {2\left| {V_{i} } \right|\left| {Y_{ii} } \right|\sin \theta_{ii} + \sum\nolimits_{\begin{subarray}{l} k = 1 \\ k \ne i \end{subarray} }^{n} {\left| {V_{k} } \right|\left| {Y_{ik} } \right|\sin \left( {\theta_{ik} + \delta_{k} - \delta_{i} } \right),} } & {i = k} \\ { - \left| {V_{i} } \right|\left| {V_{k} } \right|\left| {Y_{ik} } \right|\sin \left( {\theta_{ik} + \delta_{k} - \delta_{i} } \right), } & {i \ne k} \\ \end{array} } \right.$$

In transmission systems, the resistance is very low compared to reactance, and this makes the impedance angle close to 90. A small change in the power angle makes the $\sin \left( {\delta_{k} } \right) \cong 0$ and $cos \cong 1$. This assumption decouples the active and reactive power flow equations.

### Exchange of tie line power

The transportation of bulk reactive power over long transmission lines can be minimised only if the reactive power requirement is locally met within a transmission utility. Typically, large-sized transmission utilities have more than 10,000 substations. So, planning reactive power compensating devices like capacitors, static var compensations, dynamic compensation, and reactors at a few substations is a very critical aspect for an overall improvement of voltage response with minimal size of compensating devices.

Reactive power is consumed at load sites and should be supplied locally. The load profile continuously varies due to daily and seasonal changes. This puts stress on the grid operator to balance active and reactive power demand and often results in low or high-voltage areas^35^. The voltage gradient causes a significant transfer of reactive power across transmission lines. The upstream effects of loads lead to excessive withdrawal of reactive power over inter-utility transmission links. The change in reactive and active power flow injection at any *ith*bus during the iterative process is given in Eqs. (8–9), as follows:

8a $$\left( {\Delta Q_{tie}^{r + 1} } \right)_{i} = \left( {Q_{tie}^{0} } \right)_{i} - \sum\nolimits_{k = 1}^{n} {|V_{i} \left| {\left| {V_{k} } \right|} \right|Y_{ik} |\sin \left( {\theta_{ik} + \delta_{k} - \delta_{i} } \right)}$$

8b $$\left( {\Delta Q_{tie}^{r + 1} } \right)_{i} = \left( {Q_{tie}^{0} } \right)_{i} - \left( {Q_{tie}^{r} } \right)_{i}$$

9a $$\left( {\Delta P_{tie}^{r + 1} } \right)_{i} = \left( {P_{tie}^{0} } \right)_{i} - \sum\nolimits_{k = 1}^{n} {|V_{i} \left| {\left| {V_{k} } \right|} \right|Y_{ik} |cos\left( {\theta_{ik} + \delta_{k} - \delta_{i} } \right)}$$

9b $$\left( {\Delta P_{tie}^{r + 1} } \right)_{i} = 0$$

**Fig. 1:**
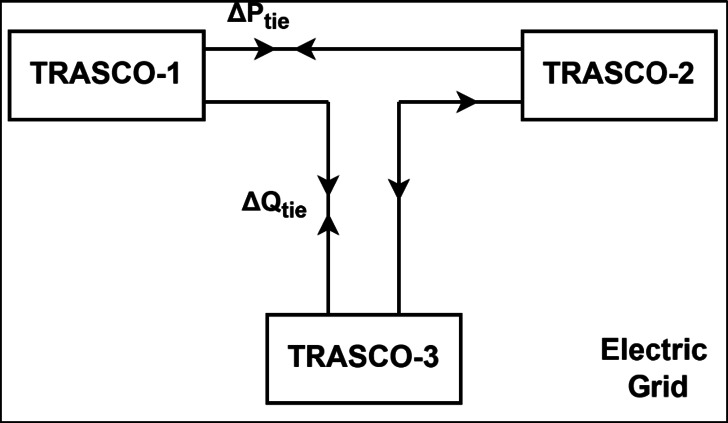
Inter TRANSCO tie line flow.

The proposed method is intended to maintain minimal change in reactive power from their scheduled value without any change in the schedule of active power while solving the power flow problem. Figure 1 illustrates the flow of change in reactive power on tie lines of different TRANCOS. Three Transco are shown in Fig.1. As depicted in Fig.1, the unscheduled flow of active and reactive power is not desired by grid operators. As per grid regulation, the change in schedule active power leads to frequency change and reactive power leads to voltage control issues, artificial loading of the tie line and high active loss in the electric grid.

### Short circuit strength

Electric grids generally have large dimensions in terms of generating units, lines, transformers, etc. The modelling of electric grids for unbalanced faults^28^ is a great challenge due to the limited information about grounding and transformer connections. However, the positive sequence impedances are readily available and balance the three-phase fault current $\left( {I_{grid}^{^{\prime\prime}} } \right)$ at a particular substation in the grid can be easily simulated in an offline simulation software. Thevenin’s equivalent impedance can be derived as Eq. (10) for a known value of $z_{f}$. A short circuit strength index $\gamma_{i}$ for $ith$ Bus is derived.

10a $$I_{grid}^{^{\prime\prime}} = \frac{{U_{n} }}{{x_{d}^{^{\prime\prime}} + z_{f} }}$$

10b $$SCR = \frac{{U_{n} }}{{P_{n} }} = \frac{1}{{\left( {x_{d}^{^{\prime\prime}} } \right)}} = \gamma_{i}$$

**Fig. 2:**
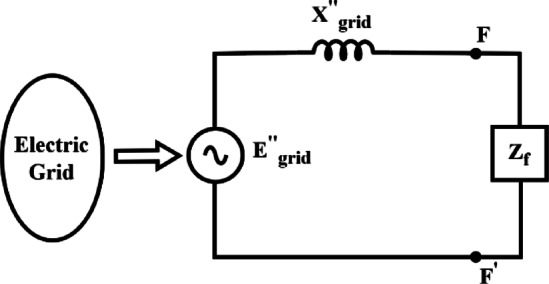
Short circuit model of electric grid.

The Thevenin equivalent of a substation short circuit model is given in Fig. 2. The higher value of $x_{d}^{^{\prime\prime}}$ Leads to a higher system voltage drop and makes it more vulnerable to voltage fluctuation. The location with a low SCR below a critical value can be considered a potential location.

The buses with a poor value of ${{\boldsymbol{\gamma}}}_{{\boldsymbol{i}}}$ are identified as candidates for the location for installing compensating devices for the local supply of reactive power.

### Q–V sensitivity index

Installation of compensating devices based on $\gamma_{i}$ The index discussed above is not very effective in radial transmission systems due to their limited voltage improvement. The performance of the proposed algorithm is further enhanced by incorporating a reactive voltage (Q–V) sensitivity index. The Q–V sensitivity analysis has been carried out by injecting a unit of reactive power. $\Delta Q_{i}$ at candidate load bus locations as listed in Eq. (11).

11a $$\Delta V_{i} = \frac{{\partial V_{i} }}{{\partial Q_{t} }}\Delta Q_{t}$$

11b $$\left( {\begin{array}{*{20}c} {\begin{array}{*{20}c} {\Delta V_{i} } \\ {\Delta V_{2} } \\ \end{array} } \\ \vdots \\ {\Delta V_{n} } \\ \end{array} } \right) = \left( {\begin{array}{*{20}c} {\begin{array}{*{20}c} {a_{11} } & {a_{12} } \\ {a_{21} } & {a_{21} } \\ \end{array} } & \ldots & {\begin{array}{*{20}c} {a_{1n} } \\ {a_{2n} } \\ \end{array} } \\ \ldots & \ldots & \vdots \\ {\begin{array}{*{20}c} {a_{n1} } & {a_{n2} } \\ \end{array} } & \ldots & {a_{nn} } \\ \end{array} } \right)\left( {\begin{array}{*{20}c} {\begin{array}{*{20}c} {\Delta Q_{1} } \\ {\Delta Q_{2} } \\ \end{array} } \\ \vdots \\ {\Delta Q_{n} } \\ \end{array} } \right)$$

11c $$\sum\nolimits_{i = 1}^{n} {|\Delta V_{i} | = \sum\nolimits_{c = 1}^{n} {\sum\nolimits_{i = 1}^{n} {a_{ic} \Delta Q_{c} } } }$$

11d $$\sum\nolimits_{c = 1}^{t} {\beta_{c} } = \sum\nolimits_{c = 1}^{t} {\sum\nolimits_{i = 1}^{n} {a_{ic} } } = \frac{{\mathop \sum \nolimits_{i = 1}^{n} |\Delta V_{i} |}}{{\mathop \sum \nolimits_{c = 1}^{t} \Delta Q_{c} }}$$

The variation in $\Delta V_{i}$ for various load buses is calculated as the modules’ aggregated sum voltage variation. The $\beta_{c}$ Sensitivity index, as the ratio of the aggregated sum of bus voltage variation against the injected reactive power, is calculated as listed in Eq. (11). The higher value of $\beta_{c}$ indicated that the candidate bus has a better impact on the improvement of the voltage profile.

### Reactive power management optimization problem

The grid operator schedules the flow of active power to match the load requirement of distribution systems. Active power flow is accompanied by unscheduled reactive power based on the system’s voltage profile. This power flow is nonlinear as discussed in Eqs. (1 and 2). The flow of reactive power over intra- and inter-region tie lines in a Large-sized National Grid is shown in Fig. 1. This research article proposes an algorithm for the minimisation of reactive power flow over the tie lines without changing the scheduled active power planned by the grid operator.

Therefore, the minimisation of the reactive power problem is formulated as a nonlinear optimisation problem. The objective function of problem formulation is given in Eq. (12), which has three components to minimise the exchange of change in reactive power. The objective function is minimised under a set of location constraints. Locations are identified for injection of optimum reactive power injection in sensitive locations. The sensitive buses are selected through a $HVGSSI$ formulated as a combination of high voltage sensitivity and weakness in short circuit buses. Equation (12) has three components; the first component is the summation change in the tie line reactive power exchange for “*t*” lines. The 2nd component is the summation voltage sensitivity index for bus voltage improvement when reactive power is injected into high-sensitivity “*k*” buses. The third component is the grid strength index, which looks into the injection of reactive power at highly weak *k* buses. The 2nd and 3rd components of Eq. (12) impose the combined set of constants termed as $HVGSSI$ for penalties generation during the optimisation process. These penalties are generated when the Q is being injected at low voltage sensitivity and high SCR buses. When the values of penalties generated become zero, that ensures the Q-being injection at those buses that jointly highly voltage sensitivity and are strong in SCR. The limits on these indices are identified based on the number of candidate buses identified for the location of reactive power support devices. The ${k}_{1}$, ${k}_{2,}$ and ${k}_{3}$ are weights assigned during solving Eq. (12).

12 $$f\left( {V_{i} ,V_{k} ,\beta_{c} } \right) = min \cdot \left\{ {k_{1} \sum\nolimits_{t = 1}^{t} {\left( {\frac{{\left( {\Delta Q_{tie}^{r + 1} } \right)}}{{Q_{tie}^{0} }}} \right)_{t} } + k_{2} \sum\nolimits_{c = 1}^{k} {\left( {\beta_{c} > \left( {\beta_{c} } \right)_{crt} } \right) + k_{3} } \sum\nolimits_{i = 1}^{k} {\left( {\gamma_{i} > \left( {\gamma_{i} } \right)_{crt} } \right)} } \right\}$$

**Fig. 3:**
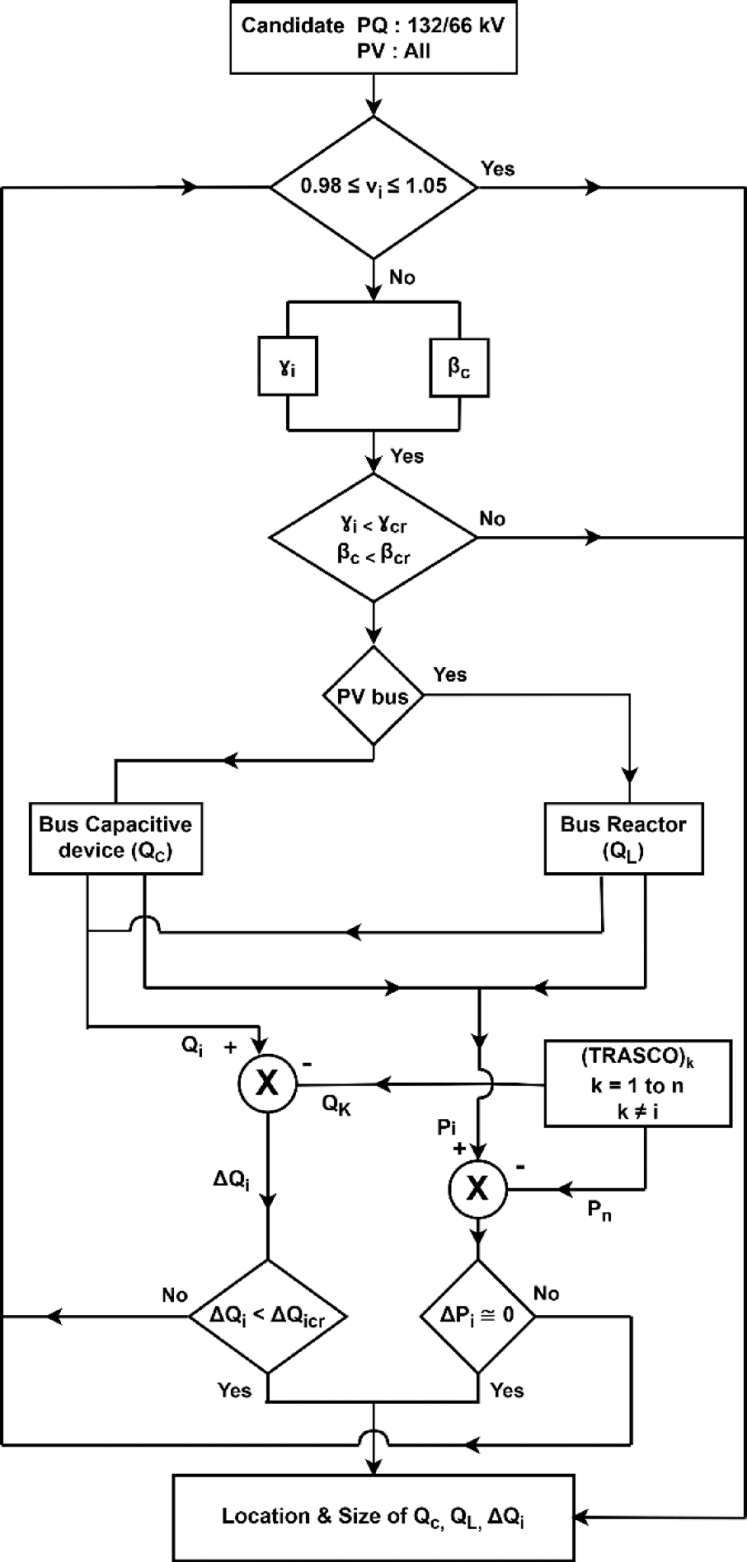
Proposed algorithm for minimisation of tie line import.

The step-by-step procedure for minimising ∆*Qtie* is shown in Fig. 3. Modelling a large-scale electric grid is challenging due to limited data availability, especially from the distribution side. Therefore, the distribution system’s load is represented as a lumped load at 132/33/11 kV substations, where the distribution system sources power from the transmission utilities. These load buses are potential sites for identifying reactive power support needed downstream, typically at 33/11 kV buses. Additionally, all PV buses are potential locations for placing reactors to control voltage if the generating unit’s exciter fails to regulate the machine’s terminal voltage. If the candidate bus voltages are below 0.98 p.u. or above 1.05 p.u., the β*c* and γ*i* indices are calculated according to Eqs. (10, 11) at those buses. Buses with β*c* and γ*i* exceeding their critical values are further considered as candidates for installing compensating devices, either at voltage control PV and PQ load buses.

The minimisation of $\Delta Q_{i}$ is performed under the constraint of $\Delta P_{i} \cong 0.$ Neighbouring transmission utilities, as proposed in Eq. (12), will be performed. The critical value of $\left( {\beta_{c} } \right)_{crt}$ and $\left( {\gamma_{c} } \right)_{crt}$ for a specified voltage is identified so that the optimal quantum of reactive injection may be placed at highly sensitive locations for overall improvement of the voltage profile and minimal import of $\Delta Q$ from intra and inter-tie lines in a large-scale National Grid.

The outcome of the above algorithm is the identification of the number of locations for each voltage level with the quantum of reactive power and reduction in the quantum of reactive power import on the tie lines.

## Simulation & result

The performance of the proposed algorithm is investigated for the Northern region of the Indian Grid. The Northern region of the Indian Grid has seven states, namely Rajasthan, Uttar Pradesh, Haryana, Punjab, Himachal Pradesh, and Jammu and Kashmir. The total active load in the Northern region was 61,707.7 MW against available power generation of 47,090.2 MW as of 11.07.2018 00:45 HRS. The size of the Northern region is large, and it involves around 9000 at the 33/132/220/400/765 kV level. It is observed that there are a few low-voltage zones where voltage is in the range of 0.85 to 0.97 p.u., especially in the distribution networks of Punjab, Haryana, Uttar Pradesh, and Rajasthan. Contrary to this, there are high-voltage regions in Himachal Pradesh and Jammu and Kashmir, where remote hydel power plants are in operation. The low voltage profile pockets in these distribution systems have resulted in the excessive drawal of reactive power from neighbouring interstate lines and inter-regional power transmission lines. The above proposed algorithm is executed in three steps. In step 1. Q–V sensitive index is calculated for all the candidate buses using Eqs. (11). In Step 2, the grid strength index is calculated for all buses. In Step 3, minimisation of inter- and intra-region tie line reactive power flow is obtained for the optimal quantum of compensation recommended on the buses that are common in Steps 1 and 2. The detailed results are listed below:

### Result of Q–V sensitivity analysis

The Q–V sensitivity analysis, as described in Eq. (11), was performed for all candidates in the Northern region of the Indian grid. The $\beta_{c}$ values are derived for a 1% change in bus voltage caused by reactive power injection at different buses. Lower voltage buses tend to have higher $\beta_{c}$ values compared to higher voltage buses. For a 33 kV bus, a voltage increase from 92 to 93% results in a $\beta_{c}$ of 0.00115. The variation of bus voltage and $\beta_{c}$ values for other buses is shown in Table 2. As indicated, $\beta_{c}$ displays a nonlinear relationship with bus voltage at a fixed reactive power injection and load. It is also observed that the $\beta_{c}$ falls as the bus voltage increases.

**Table 2:** $\beta_{c}$ index for different voltage ranges.

Voltage range	Bus voltage		
	33	132	220
0.85–0.86	0.00175	0.0014875	0.00105
0.86–0.87	0.00163	0.0013855	0.000978
0.87–0.88	0.00149	0.0012665	0.000894
0.88–0.89	0.00144	0.001224	0.000864
0.90–0.91	0.00127	0.0010795	0.000762
0.91–0.92	0.0012	0.00102	0.00072
**0.92–0.93**	**0.00115**	0.0009775	0.00069
0.93–0.94	0.0011	0.000935	0.00066
0.94–0.95	0.0009	**0.000765**	0.00054
0.95–0.96	0.00078	0.000663	0.000468
0.96–0.97	0.00056	0.000476	**0.000336**
0.97–0.98	0.00045	0.0003825	0.00027
0.98–0.99	0.00036	0.000306	0.000216
0.99–0.100	0.0002	0.00017	0.00012
1.0–1.01	0.00015	0.0001275	0.00009
1.01–1.02	0.00025	0.0002125	0.00015
1.02–1.03	0.00045	0.0003825	0.00027
1.03–1.04	0.0008	0.00068	0.00048
1.04–1.05	0.00101	0.000858	0.000606

Significant values are in bold.

The system under consideration is big, and therefore, the ${\mathrm{V}}_{\mathrm{avr}}$ It is considered a parameter for investigating the performance of the proposed algorithm. The load on the distribution side is lumped at 33/132/220 kV buses; it is derived that way. ${\mathrm{V}}_{\mathrm{avr}}$, at 33 kV buses, is 0.932%. ${\mathrm{V}}_{\mathrm{avr}}$ values for other buses are provided in Table 3. A 0.11% average increase in bus voltage is recorded for a unit reactive source injection at 33 kV. The % improvement of other buses is also listed in the Table 3. The ${\left({\beta }_{c}\right)}_{crt}$, the value is determined for each voltage level based on the ${\mathrm{V}}_{\mathrm{avr}}$ value and is highlighted in bold in Table 2. Buses whose β*c* is below ${\left({\beta }_{c}\right)}_{crt}$ are segregated out as candidate locations for the installation of reactive power compensating devices for Stage 2 of the proposed algorithm.

**Table 3:** Average voltage profile in the northern region.

kV	765	400	220	132	33
Number.	54	72	579	5382	7133
$V_{avr}$	0.991	0.985	**0.960**	**0.945**	**0.932**
$V_{avg}^{imp}$	1.005	1.004	0.995	0.984	0.965
% buses	–	–	22.8	35.5	43.2
%$V_{avr}$	1.32	1.87	3.60	4.08	3.57

Significant values are in bold.

The percentage of buses chosen based on $\beta_{c}$ is listed in Table 3. It is observed that approximately 43.2% of the 7133 buses at 33 kV are selected as candidate buses for stage 2 of the proposed algorithm. The percentages for other voltage levels are also included, illustrating a decrease as system voltage increases due to lower $\beta_{c}$ values. Final ${\mathrm{V}}_{{{\mathrm{avg}}}}^{{{\mathrm{imp}}}}$ for various bus voltages after installation of reactive power compensating devices is also listed in Table 3.

### Result of grid strength analysis

Extensive short circuit studies are performed to assess the grid strength for the Northern region of the Indian grid in the PSSE simulation software. The Thevenin’s equivalent, as shown in Fig. 2, for various voltage levels has been derived using the grid short circuit ratio as listed in Eq. (10) above. Based on the voltage levels, the buses are classified as strong, moderate, and low. It is observed that around 64.2% of 7133 buses at 33 kV are classified as weak buses. However, very few buses are classified as a strong category. The percentage of weak buses decreases as the bus location moves towards grid short-circuit sources. The % number of buses grouped based on grid strength for the NR region of the Indian grid is given in Table 4.

**Table 4:** $\gamma_{i}$ for various grid strength.

	Strong	Moderate	Weak
$x_{d}^{^{\prime\prime}}$(p.u)	> 3.5	1.5 ≤ ${ SCR}_{i}$ ≤ 3.5	1.5 <
$\gamma_{i}$	0.2851 <	0.2851 ≤ ${SCR}_{i}$ ≤ 0.7	> 0.7
765 kV	77.8	22.2	0.0
400 kV	30.6	52.8	16.7
220 kV	23.3	43.9	32.8
132 kV	0.7	33.2	66.2
33 kV	0.3	35.5	**64.2**

Significant values are in bold.

**Fig. 4:**
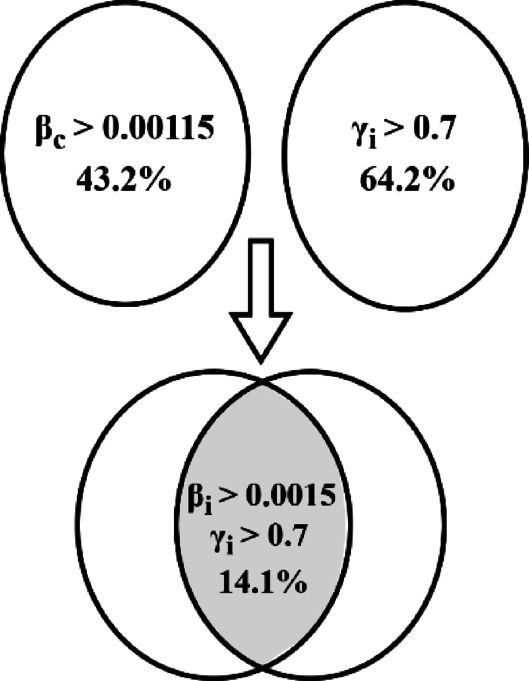
Summary of sensitive buses.

In the above step 1 of the proposed algorithm, 43.2% of buses at 33 kV are identified as high Q–V sensitivity buses. On the other hand, 64.2% of 33 kV buses are identified as candidate locations for injecting Q support. The installation of Q-support devices on such a large number. Of buses is not practically feasible due to the availability of space, cost, and operational constraints. So, further optimisation of candidate location is performed by the union of both the above-discussed indices. Figure 4 summarises the details of buses with their $\left( {\beta_{c} } \right)_{cr}$ and $\gamma_{i}$ identified as the final location of Q-support devices.

It is concluded that there are 14.1% of buses at 33 kV are promising buses identified for Q-injection where $\beta_{c}$ is higher than $\left( {\beta_{c} } \right)_{cr}$ and $\gamma_{i}$ is greater than 0.7 $\left( {\left( {\gamma_{c} } \right)_{crt} } \right)$.

### Optimization reactive power management

The power flow of the Northern grid of the Indian Grid is performed in the PSSE simulation software. The power flow in the NR region without proposed compensating devices is considered the base case. The results for the base case are listed in Table 5. The total load in the active load is 62,135 MW, which is imported from the Northern region generation of 47,951 MW and the import of 16,152 MW from the neighbouring region of the Indian grid. The reactive power is imported from generating sources, line charging, and absorbed in the system. So, management of reactive power is very tricky.

**Table 5:** Power flow summary in northern region.

Element	Base case		Proposed algorithm	
	P (MW)	Q (MVAr)	P (MW)	Q (MVAr)
Generation	47,951	10,304	47,951.4	− 686.92
Load	62,135	17,594	62,139.1	19,595
Shunt inductor	0	11,048.8	0	2043.1
Line inductive effect	0	25,284.3	0	26,053.5
Line charging effect	0	68,778.1	0	71,194.5
Active power losses	1968	0	1810.6	0
Reactive system demand	0	26,747	0	25,200
Tie-Line −import/ + export	− 16,152	− 1592.2	− 15,999	− 383

In the base case, as mentioned above. There is an import of 1592.2 MVAr over tie lines from outside the northern regions. The supply and absorption of reactive power from line effect, bus shunt reactors, and system demand are mentioned in Table 5. This high import of reactive power from outside the NR region causes high reactive power flow over the inter-utility lines in the NR region. This leads to artificial loading of inter-regional and intra-regional tie lines, and power transfer capability is decreased. Collaterally, this will also cause high active power loss in the grid.

The proposed algorithm, as listed above, identifies the quantum and location of reactive power-compensating devices. The buses, which are more sensitive than ${\left({\beta }_{c}\right)}_{cr }$ and the grid short circuit strength is more than ${\left({\gamma }_{c}\right)}_{crt,}$ are classified as a final bus for the location of compensating devices. The % of buses at the final location for each voltage location is identified. The proposed algorithm identifies 9421.8 MVAr to be installed at different voltage levels of each utility grid in the NR of the Indian Grid. It is observed that the proposed algorithm identified around 14.1% of buses out of 2310 buses for installation of Q. Similarly, for other voltage levels, the % of finalised candidate buses is listed in Table 6.

**Table 6:** Identified compensation on the % bus.

Utility	Q (MVAr)	% Bus location		
		33 kV	132 kV	220 kV
Punjab	1024.4	16	21	9
Haryana	1541.6	13	23	6
Rajasthan	1649	15	26	14
Delhi	1465	9	11	6
Uttar Pradesh	2774	18	26	16
Uttrakhand	600	15	19	11
Himachal	37.6	16	15	8
J and K	330.2	14	11	2
Chandigarh	0	11	8	0
Total	9421.8	14.1	17.8	8.0

**Fig. 5:**
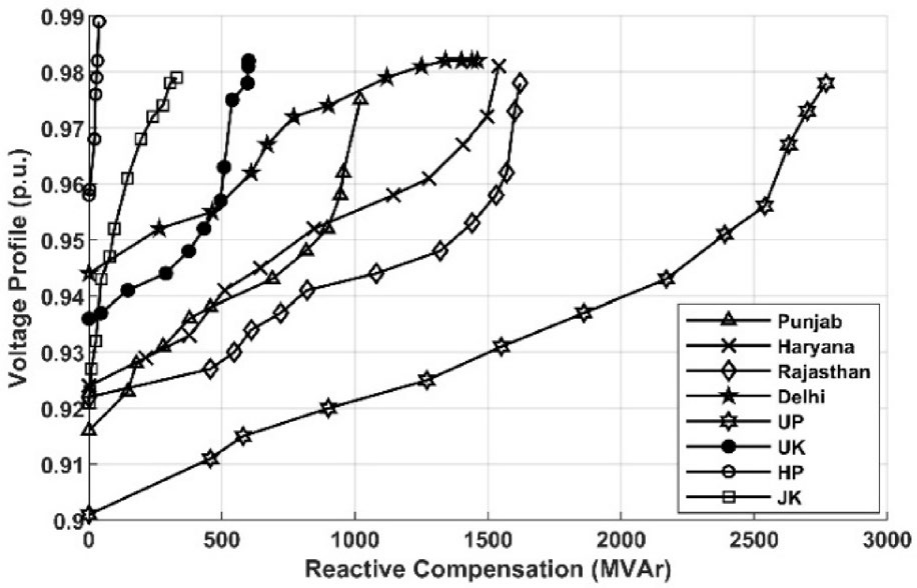
Performance of Q-injection on systems voltage.

The improvement of the average voltage in p.u for Q-injection at highly sensitive buses, as identified above, is shown in Fig. 5. It indicates the system’s voltage improves from around 0.9 p.u. to 0.98 p.u., holding for various transmission utilities in the northern region. The improvement in system voltage for Q-injection is nonlinear due to the behaviour of the load and characteristics of the power system.

The proposed algorithm is solved with the help of a fast and global minima-based DSA. The optimised power flow in the NR-region is listed in Table 6. It is observed that the import of reactive power over inter-regional tie lines is minimised from 1592,2 MVAr to 383 MVAr for the same load of 62,135 MW being fed by Transmission utilities.

The optimised flow of reactive power through the proposed algorithm for UP TRANSCO is given in Table 7. It is observed that in the base case, there was an import of 4421 MVAr from the neighbouring utilities in the NR-region, and now that has been minimised to 2079 MVA. Similarly, a comparative analysis for the optimised flow of reactive power with the proposed algorithm based on other utilities in the NR-region is listed in Table 8. It is found that in most cases, there is an average 48.4% decrease in the import of reactive power from other neighbouring transmission utilities in the NR-region of the Indian grid.

**Table 7:** Optimised reactive power flow in UP TRANSCO.

FROM	TO	Q(MVAR)	Q(MVAR)
UTTAR PRADESH	HARYANA	81	63
	RAJASTHAN	29	− 33
	DELHI	− 15	− 11
	UK	− 184	− 100
	NR_ISTS_DEL	− 59	− 85
	NR_ISTS_UP	− 2953	− 1885
	NR_ISTS_UTT	2	− 18
	NR_ISTS	− 451	− 119
	SINGRAUL	− 469	− 373
	DADR-NCR	− 172	− 80
	UNCHAHAR	40	156
	AURAIYA	− 67	− 31
	NAPS	− 73	128
	TANAKPUR	16	11
	DHAULIGA	− 10	8
	ROSA	− 126	103
	JHARKHAND	− 31	− 31
	ER_ISTS_BIH	− 16	− 2
	WR_ISTS_MP	37	220
	Total	− 4421	− 2079

**Table 8:** Optimised reactive power flow in northern region.

Exchange of MVAr			
	Base case	Proposed algorithm	% change
Punjab	− 1520	− 1162	− 23.6
Haryana	− 1769	− 1500	− 15.2
Rajasthan	− 829.1	7	− 100.8
Delhi	− 1336	− 239	− 82.1
UP	− 4421	− 2079	− 53.0
UK	− 130	25	− 119.2
HP	180	161	− 10.6
JK	− 864	− 729	− 15.6
Total	− 10,689.1	− 5516	− 48.4

**Fig. 6:**
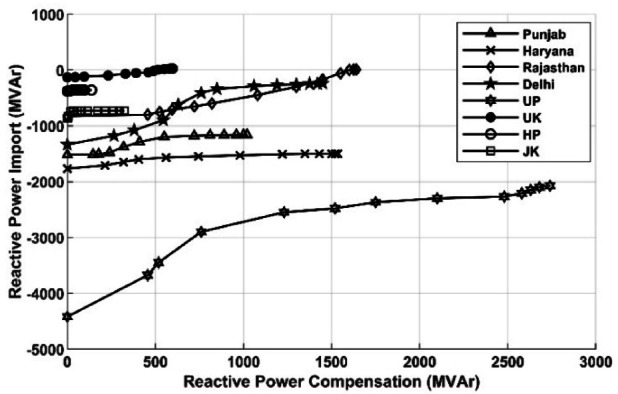
Impact of Q-injection on reactive import on inter-utility lines.

The fall in the cumulative import of reactive power for individual transmission utilities in the NR region is depicted in Fig. 6. The variation of reactive power against reactive power compensation is also non-linear and holds a similar relation to that observed in Fig. 6.

The identification of highly sensitive compensation at the optimal location at 33/132/220 kV levels has reduced the import of reactive power in state transmission utilities, and this has finally resulted in a lesser import of reactive power from the inter-regional tie lines. Table 9 provides the details of the % change in the flow of reactive power over the inter-regional tie lines. It is observed that in the majority of lines, there is a fall in the import of reactive power, but in a few cases, there % increase due to a change in voltage profile in the NR-region. In the base case, there was an import of 1208 MVAr, and this has been reduced to 383 MVAr. So, on average, there is a 76% decrease in the import of reactive power. This significant reduction will alleviate the stress on these lines and enhance the power transfer limits. As a major outcome of this work, the heavy decrease in inter- and intra-region tie lines will avoid the heavy reactive power penalty imposed on the transmission utilities by the grid regulators.

**Table 9:** % decrease in tie lines reactive power import.

FROM AREA	TO AREA	Base case	Proposed algorithm	
		Q (MVAr)	Q (MVAr)	% change
UP	ER_ISTS_BIH	− 16	− 2	− 88
AURAIYA	MP	− 6	0	− 100
NR_ISTS	GUJARAT	− 1012	− 988	− 2
NR_ISTS_RAJ	GUJARAT	− 36	47	− 231
RAJASTHAN	MP	− 4	− 5	25
RAJASTHAN	WR_ISTS_MP	192	285	48
NR_ISTS_UP	WR_ISTS_MP	75	304	305
NR_ISTS_UP	NER-ISTS	− 366	− 366	0
UP	JHARKHAND	− 31	− 31	0
RAJASTHAN	WR_ISTS_GUJ	− 272	− 200	− 26
UP	WR_ISTS_MP	37	220	495
NR_ISTS_UP	ER_ISTS_WB	− 154	− 134	− 13
NR_ISTS_HAR	WR_ISTS_CHAT	− 1176	− 1128	− 4
RAPS-C	WR_ISTS_MP	107	135	26
TANAKPUR	FAR-WEST NEP	− 9	− 10	11
NR_ISTS_UP	ER_ISTS_BIH	1080	1490	38
TOTAL		− 1592	− 383	− 76

**Fig. 7:**
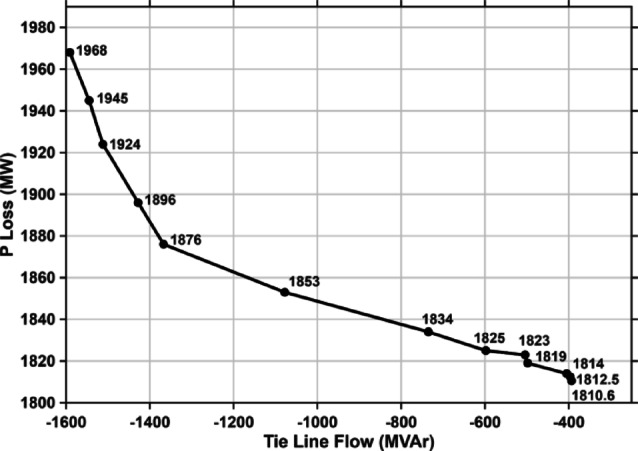
Impact of Q-injection on reactive import.

As observed in the Tables. 6, 7 and 8, a heavy reduction in the import of reactive power will decrease the artificial loading of lines and other power equipment. As mentioned above, the active load in the northern region is 62,137 MW, and the corresponding active power loss is 1968 MW. The proposed algorithm reduces active power loss to 1810.6 MW. Thus, there is around an 8% reduction in active power loss in the Northern region, as listed in Table 10. This remarkable fall in active power loss will enhance the operational efficiency and power procurement cost for power utilities. The decrease in active loss during a fall in reactive import over tie lines in NR is plotted in Fig. 7.

**Table 10:** % reduction in active power loss (MW).

Load (MW)	Base case active loss (MW)	Proposed algorithm loss (MW)	% reduction
62,135	1968	1810.6	− 7.99

The performance of Q-injection by injecting reactive power compensating devices at highly sensitive buses and their impact on the reduction of tie lines import and active losses are well established through the above discussion and result analysis.

## Conclusion

The high variation in loading profiles and inadequate planning of reactive power management create low-voltage profile areas in large electric grids. This leads to the artificial loading of inter- and intra-regional tie lines in a large electric grid, and subsequently, reactive power penalties are imposed on the transmission utilities. Mostly, power utilities install reactive power compensating devices at low voltage locations identified through operational voltage & voltage sensitivity analysis. From simulation studies, it is learnt that the locations identified through voltage sensitivity analysis are quite high, and practically the installation of reactive devices on such large locations is not feasible. Additional power utilities are penalised by regulators for uncontrolled exchange of reactive power, and no particular consideration has been given to the flow of reactive power over inter-regional tie lines in an electric grid.

In this research article, the inter-and intra-region tie-lines flow of reactive power in a large-sized electric grid is minimised through the formulation of reactive power management as a nonlinear optimisation problem.

The proposed optimisation problem is solved within constraints set generated through a $HVGSSI$. It is observed that alone voltage sensitivity index identified around 64.2% of 33 kV buses as candidates for locating the reactive power devices. Contrary to this, the grid strength sensitivity index identifies around 43.2% buses as candidate locations for reactive power support devices.

The proposed HVGSSI reduces the no. of buses to a small set of highly sensitive buses, limited to about 14.1% of total buses, that are practically feasible for the utility operators to install the reactive power support devices in their electric grid. Installation of 9421.8 MVAr at 33 kV highly sensitive locations with ${\upbeta }_{{\mathrm{c}}} > \left( {{\upbeta }_{{\mathrm{c}}} } \right)_{{{\mathrm{cr}}}}$ & ${\upgamma }_{{\mathrm{i}}} > 0.7$ results in a reduction of 76% import of reactive power from 1592 to 383 MVAr on the inter-regional tie lines in NR of the Indian grid. It is also observed that there is around an 8.0% reduction in total active power loss in the NR-region. The recommended optimal reactive injections have resulted in an average improvement of around 3 to 4% in the system’s voltage profile at 33 kV buses in the NR-region. The reactive power requirement identified through the minimisation of tie line reactive power flow based on HVGSSI has been implemented in the NR-region of the Indian grid.

The proposed algorithm serves as a guiding method for large power utilities, where selecting candidate buses is generally difficult because it relies on the voltage sensitivity index, and little attention has been given to controlling reactive power exchange among connected utilities. In this context, the proposed algorithm is very effective in identifying the minimum reactive power sources needed at fewer buses to reduce the large import of reactive power over intra- and inter-regional tie lines in the NR-region of the Indian grid.

The proposed problem is not extended to the renewables-rich electric grids. These generating resources will impose the additional constraints set due to their limited reactive power generation capacity as compared to conventional synchronous generating resources. The presented work will be a reference for researchers to explore the reactive power management challenges in electric grid hosting large-scale solar and wind power plants, along with grid-connected battery energy storage systems.

## Data Availability

The datasets used and/or analysed during the current study are available from the corresponding author upon reasonable request.

## Abbreviations

$P_{i}$: Active power injection at $ith$ bus
$Q_{i}$: Reactive power injection at $ith$ bus
$$V_{i}$$: $$ith$$ Bus voltage
$$V_{k}$$: $$kth$$ Bus voltage
$Y_{ik}$: Admittance magnitude between $ith$ and $kth$ bus
$\theta_{ik}$: Admittance angle between $ith$ and $kth$ bus
$$\delta_{i}$$: Angle of $$V_{i}$$
$$\delta_{k}$$: Angle of $$V_{k}$$
$(Q_{tie}^{0} )_{i}$: Initial reactive power flow
$\Delta Q_{tie}^{r + 1}$: Change in reactive power during $i + 1th$ iteration
$(P_{tie}^{0} )_{i}$: Initial active power flow
$\Delta P_{tie}^{r + 1}$: Change in active power during $i + 1th$ iteration
$I_{grid}^{^{\prime\prime}}$: Sub transient fault current
$$U_{n}$$: Prefault voltage
${\mathrm{V}}_{{{\mathrm{avr}}}}$: Average bus voltage
${\mathrm{V}}_{{{\mathrm{avg}}}}^{{{\mathrm{imp}}}}$: Improved bus voltage
$x_{d}^{^{\prime\prime}}$: Sub-transient reactance
$$z_{f}$$: Fault impedance
$\gamma_{i}$: Grid short circuit index
$Q-V$: Reactive power–voltage
$\frac{{\partial V_{i} }}{{\partial Q_{t} }}$: $ith$ Voltage change for reactive power injection at $kth$ Bus.
$\Delta Q_{t}$: Unit compensation reactive power
$a_{nn}$: Voltage bus sensitivity index.
$$\beta_{c}$$: Q–V sensitivity index
$\left( {\beta_{c} } \right)_{crt}$: Critical value of $\beta_{c}$
$\left( {\gamma_{c} } \right)_{crt}$: Critical value of $\gamma$
$HVGSSI$: Hybrid voltage-grid strength sensitivity index
*VAR*: Volt ampere reactive
K: Number of buses for $\gamma_{i}$ index
